# Diagnosis of pulmonary leiomyosarcoma extending into the main bronchus using repeated transbronchial cryobiopsy

**DOI:** 10.1002/rcr2.1078

**Published:** 2022-12-21

**Authors:** Shinya Tsukamoto, Takamasa Kitajima, Satoshi Marumo, Motonari Fukui

**Affiliations:** ^1^ Respiratory Disease Center Tazuke Kofukai Medical Research Institute, Kitano Hospital Osaka Japan

**Keywords:** lung neoplasms, proton therapy, pulmonary leiomyosarcoma, transbronchial biopsy, transbronchial cryobiopsy

## Abstract

The diagnosis of pulmonary leiomyosarcoma using bronchoscopy is difficult, and surgical resection is often performed for definitive diagnosis and curative therapy. We report a case of pulmonary leiomyosarcoma, successfully diagnosed using repeated transbronchial cryobiopsy (TBCB). A 69‐year‐old‐woman was found to have an oval mass in the left hilar region extending into the left main bronchus on computed tomography (CT). All transbronchial biopsy specimens were necrotic, but repeated TBCB removed the necrotic tissue from the tumour and finally led to the diagnosis of pulmonary leiomyosarcoma. Proton therapy was administered, which caused shrinkage of the tumour. Thus, TBCB is useful for definitive diagnosis of leiomyosarcoma without surgical biopsy. Repeated TBCB can reduce tumour volume, eliminate atelectasis, and reduce the extent of radiotherapy exposure.

## Graphical abstract

Pulmonary leiomyosarcoma is a rare tumour. Diagnosis by bronchoscopy is difficult because of significant necrotic tissue in the tumour and laborious exfoliation of the cells. We report a case of pulmonary leiomyosarcoma, successfully diagnosed using repeated transbronchial cryobiopsy (TBCB).

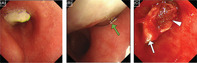

## INTRODUCTION

Pulmonary leiomyosarcoma is a rare tumour that accounts for 0.5% of all lung malignancies.^1^ The prognosis is poor, with a median overall survival of 33 months in operated cases and 4 months in inoperable cases.^1^ Diagnosis by bronchoscopy is difficult because of significant necrotic tissue in the tumour and laborious exfoliation of the cells. Hence, surgery is often performed for diagnosis. Transbronchial cryobiopsy (TBCB) is a technique wherein the target tissue is quickly frozen to obtain a larger sample with superior architectural preservation and minimal crush artefacts.^2^ It is often used to diagnose interstitial lung disease. Here, we report a case of pulmonary leiomyosarcoma diagnosed using repeated TBCB.

## CASE REPORT

**FIGURE 1:**
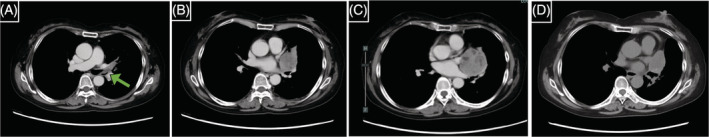
(A, B) CT scans at initial examination show a white mass extending into the left main bronchus from the bronchial orifice of the left upper lobe (green arrow). (C) A CT scan just before proton therapy shows further tumour growth. (D) A CT scan for 8 months post‐proton therapy shows a gradually shrinking tumour

**FIGURE 2:**
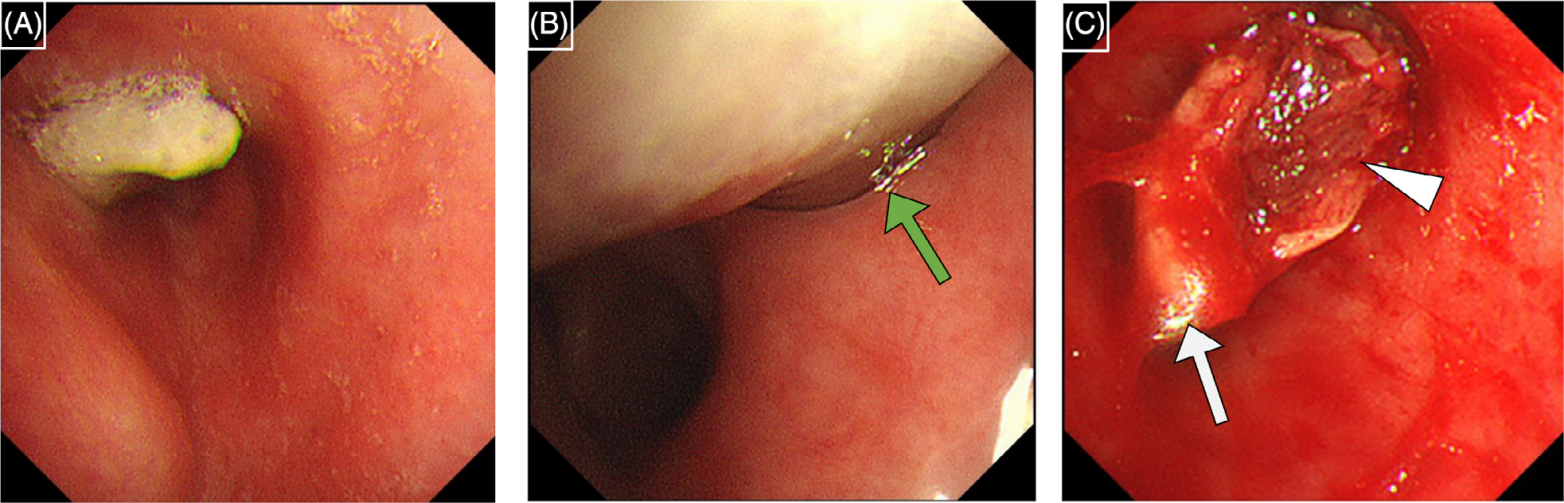
(A, B) Bronchoscopic images at initial examination show a white mass extending into the left main bronchus from the bronchial orifice of the left upper lobe (green arrow). (C) During bronchoscopic re‐examination, slight reddish appearance with blood oozing at the biopsy seemed non‐necrotic tissue (white arrowhead), unlike necrotic tissue, which appears white (white arrow)

**FIGURE 3:**
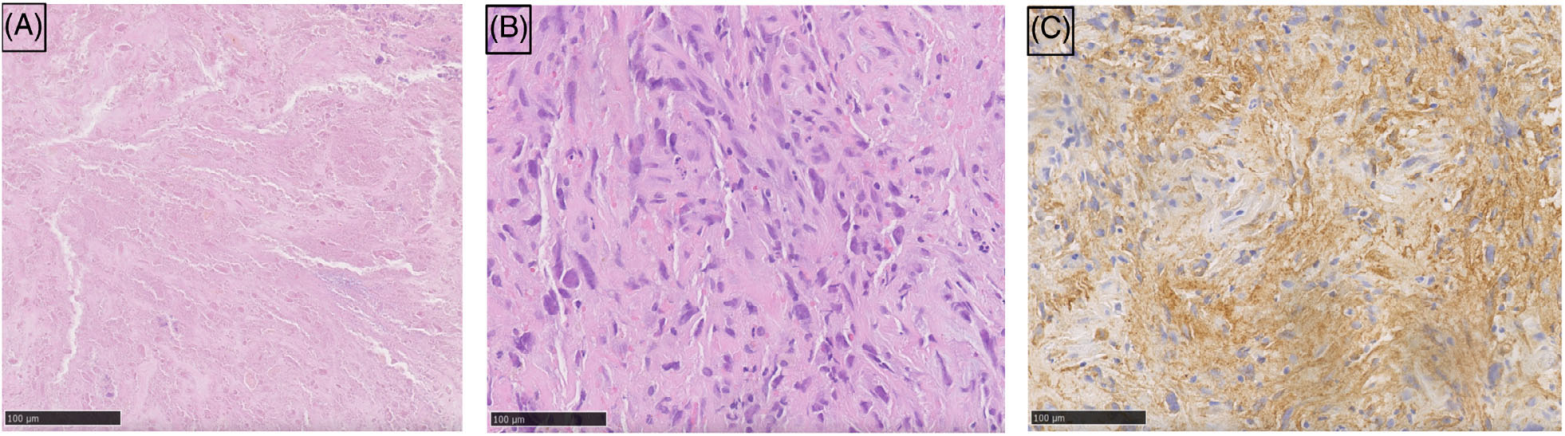
Specimens obtained during the first bronchoscopy were composed of coagulative necrotic tissue (A). Haematoxylin‐eosin staining of the tumour shows spindle and round‐shaped atypical cells with eosinophilic cytoplasm (B). Immunohistochemistry results are positive for alpha‐smooth muscle actin (C). (Original magnification: ×200)

A 69‐year old woman presented with a 2‐week history of cough and dysponea. Computed tomography (CT) revealed a 35 × 32 × 39 mm oval mass in the left hilar region, extending into the left main bronchus (Figure 1A, B). We performed the bronchoscopy and found a white mass extending into the left main bronchus from the bronchial orifice of the left upper lobe (Figure 2A, B). All specimens obtained from transbronchial biopsy (TBB) were composed of coagulative necrotic tissue (Figure 3A). Two weeks later, CT was repeated, and the scan showed that the mass had increased to 50 × 42 × 52 mm. Atelectasis was observed in the lingular segment of the left lung. Bronchoscopic re‐examination revealed an extension of the mass to the vicinity of the tracheal bifurcation. First, we performed TBCB 10 times to collect the tissues present away from the tumour centre. At this time, the tumour appeared white without blood oozing, suggesting that it should be all necrotic. Subsequently, we carried out additional TBCB and TBB for a total of 23 times to obtain the specimens of tissues located near the tumour centre. As the examination progressed, it became possible to confirm the slight reddish surface and blood oozing to some degree during the biopsy (Figure 2C), so we considered that we had taken sufficient viable tissues. The former specimens were all necrotic, but the latter showed spindle‐shaped and round‐shaped atypical cells with eosinophilic cytoplasm (Figure 3B). Immunohistochemical analysis was positive for alpha‐smooth muscle actin (Figure 3C) and HHF35, but negative for AE1/AE3, TTF‐1, p40, CK5/6, and CD34, leading to a diagnosis of leiomyosarcoma. Neither metastases nor other primary sites were found after positron emission tomography‐CT with 18F‐fluorodeoxyglucose. Thus, the patient was diagnosed with primary pulmonary leiomyosarcoma. Surgery with total lung resection was difficult due to vascular invasion. Since there is little evidence of application of chemotherapy and radiation therapy in primary pulmonary leiomyosarcoma, we administered proton therapy at a total dose of 66 Gy in 25 fractions over a period of 8 weeks to achieve a strong local therapeutic effect. The tumour gradually shrank to 43 × 27 × 37 mm for 8 months post‐proton therapy, along with atelectasis resolution (Figure 1D).

## DISCUSSION

We report a case of pulmonary leiomyosarcoma that was successfully diagnosed using repeated TBCB. To the best of our knowledge, this is the first report on the usefulness of TBCB for the definitive diagnosis of pulmonary leiomyosarcoma. Previously, several cases have reported that a definitive diagnosis cannot be made using TBB because of necrotic tissue. Many specimens are required to obtain viable tissue, and a considerable mechanical force is required to collect specimens from a firm tumour characterized by smooth muscle tissue. Brushing and washing tumour cells are of little value since they are difficult to exfoliate.^3^ Thus, preoperative diagnosis of pulmonary leiomyosarcoma is difficult, and surgical resection is preferred for definitive diagnosis and curative therapy.^1^ Since TBCB can obtain larger specimens with superior architectural preservation and minimal crush artefact than TBB,^2^ TBCB may be useful in the definitive diagnosis of leiomyosarcoma. In the case of a necrotic mass extending into the bronchus, leiomyosarcoma should be considered, and TBCB should be repeated.

We believe that a non‐invasive definitive diagnosis can promote non‐surgical treatment options. Surgery is the first‐line treatment for pulmonary leiomyosarcomas. Previous studies have reported prolonged overall survival in surgical cases compared with nonsurgical cases.^1^ Chemotherapy and radiation therapy are the usual first‐line treatments for inoperable patients. However, there is a paucity of data on their effectiveness, as well as proton therapy, which is another treatment option for inoperable patients. Nevertheless, several studies on proton therapy show good overall survival and focal control for unresectable malignant soft tissue sarcomas, including leiomyosarcomas.^4^ Further exploration of the effectiveness of proton therapy will be possible when more cases are definitively diagnosed using TBCB.

In addition, repeated TBCB can reduce the tumour volume, thus resolving atelectasis and reducing the area requiring irradiation. Previously, several cases of atelectasis due to the extension of pulmonary leiomyosarcomas into the bronchus were reported.^5^ This may be because leiomyosarcomas, which often originates in the bronchial or bronchiolar smooth muscle,^1^ can easily extend into the bronchus during its progression. Repeated TBCB may be useful for a part of local treatment.

We report the first case of pulmonary leiomyosarcoma that was successfully diagnosed using repeated TBCB. Repeated TBCB may also be useful in diagnosing pulmonary malignancy with significant necrosis regardless of pulmonary leiomyosarcoma. However, further accumulation of similar cases is expected to substantiate TBCB as a powerful diagnostic technique.

## AUTHOR CONTRIBUTIONS

Shinya Tsukamoto drafted the manuscript. Takamasa Kitajima, Satoshi Marumo and Motonari Fukui revised the manuscript. All authors approved the final manuscript.

## CONFLICT OF INTEREST

None declared.

## ETHICS STATEMENT

The authors declare that appropriate written informed consent was obtained for the publication of this manuscript and accompanying images.

## Data Availability

The data that support the findings of this study are available from the corresponding author upon reasonable request.
